# Pilot Cross-Platform Bench Characterization of Injectable Biostimulator Products Using a Predefined Descriptive Workflow

**DOI:** 10.3390/biomedicines14102154

**Published:** 2026-09-23

**Authors:** Wojciech Gruszczyński, Monika Filipowska, Paweł Kubik

**Affiliations:** K-LAB Badania i Rozwój, 81-312 Gdynia, Poland; monika.filipowska@k-lab.com.pl (M.F.); pawel.kubik@k-lab.com.pl (P.K.)

**Keywords:** injectable biostimulators, biomaterial characterization, poly-L-lactic acid, poly-D,L-lactic acid, polycaprolactone, calcium hydroxyapatite, particle morphology, reconstitution, light microscopy

## Abstract

**Background/Objectives:** This descriptive pilot documented preparation-related, microscopic, and aqueous-exposure appearances of nine injectable biostimulator products spanning poly-L-lactic acid (PLLA), poly-D,L-lactic acid (PDLLA), polycaprolactone (PCL), and calcium hydroxyapatite (CaHA). **Methods:** Nine products were examined (one unit per product). Five reconstitution-required products underwent manufacturer-specified dilution and mixing, a study-defined 120-min observation period, and re-agitation; this interval is not clinical preparation guidance. Four products were assessed as supplied. Following baseline sampling, the remaining material underwent product-specific introduction into dishes initially containing 15 mL of sterile 0.9% NaCl and incubation for 72 h at 37 °C. Three random fields were photographed and assessed per product, stage, and magnification. Fields were unpaired; no statistical analysis was performed. **Results:** Four reconstituted samples had a single-phase appearance and one a liquid–foam appearance; both PDLLA samples appeared visually thick/cohesive. Immediate bulk patterns comprised homogeneous dispersion (two samples), non-homogeneous dispersion (four), and retained rod-like extrusion (three). Microscopy showed spherical, ovoid, and irregular particles and spindle-like microstructures. After 72 h, dominant morphology categories corresponded to baseline, while additional appearances and aggregation categories differed between unpaired fields for some samples. **Conclusions:** The examined samples showed features not uniformly aligned with material platform. The images provide a preliminary reference for further physicochemical and in vivo histopathological studies. This single-unit study does not establish reproducibility, chemical stability, or clinical performance.

## 1. Introduction

Injectable biostimulator products used in aesthetic medicine span several biomaterial platforms, most commonly poly-L-lactic acid (PLLA), poly-D,L-lactic acid (PDLLA)-based systems, polycaprolactone (PCL), and calcium hydroxyapatite (CaHA)-based formulations [1,2,3,4]. Although these products are often clinically grouped under broad terms such as “biomaterials” or “collagen stimulators,” they are neither compositionally nor structurally equivalent. Differences in principal material chemistry, particulate or microstructural architecture, carrier phase, and manufacturer-specified preparation requirements may contribute to different early physicochemical behaviors after preparation and aqueous exposure [1,2,3,4,5,6].

For reconstitution-required products in particular, the formulation state at the time of administration is influenced not only by nominal material class but also by the preparation procedure defined in the instructions for use (IFU). Published comparative studies of polylactide fillers have documented inter-product differences in reconstitution behavior, viscosity, particle morphology, and hydrolytic behavior in aqueous media, while microscopy-based analyses have shown variability in particle size and shape among commercially available PLLA, PDLLA, and CaHA products [5,7,8,9]. These observations suggest that products grouped under a single platform designation should not be assumed to be physicochemically equivalent on the basis of class name alone.

Descriptive light microscopy can document the general appearance of particles and microstructures before their assessment in more complex experimental systems. Histopathological studies have used the morphology of filler material together with the surrounding tissue response to help distinguish implanted materials [10]. A preliminary reference of product appearances may therefore assist investigators planning tissue studies, although recognition after tissue processing and in vivo exposure requires separate evaluation.

Cross-platform studies documenting preparation-related macroscopic features, baseline microscopic appearance, and subsequent aqueous-exposure observations within one descriptive workflow remain limited [4,5,7,8]. A common reporting structure can organize observations across reconstitution-required and ready-to-use formulations while making differences in preparation, carrier, and exposure conditions explicit.

This pilot descriptive bench study documented the observable appearance and bulk behavior of nine commercially available PLLA-, PDLLA-, PCL-, and CaHA-based formulations. The predefined workflow comprised serial macroscopic observation of reconstituted products, baseline light microscopy, immediate assessment after introduction into saline, and macroscopic and microscopic reassessment after 72 h at 37 °C. Observations were organized using descriptor sets established before assessment. The study was intended as a completed descriptive stage providing a starting point for subsequent physicochemical analyses and in vivo studies, including histopathological assessment of the materials.

## 2. Materials and Methods

### 2.1. Study Products and Preparation Protocols

Nine commercially available injectable biomaterial products representing four principal material platforms were included: PLLA-1 (JULÄINE, Nordberg Medical AB, Huddinge, Sweden), PLLA-2 (Sculptra, Galderma, Lausanne, Switzerland), PLLA-3 (Lanluma, GANA R&D Co, Ltd., Seongnam-si, Republic of Korea), PDLLA-1 (Lenisna 200, VAIM Co., Ltd., Daejeon, Republic of Korea), PDLLA-2 (JUVElook, VAIM Co., Ltd., Daejeon, Republic of Korea), PCL-1 (Gouri, Dexlevo Inc., Seoul, Republic of Korea), PCL-2 (Ellansé, AQTIS Medical BV, Utrecht, The Netherlands), CaHA-1 (HArmonyCa, Panaxia, Ltd., Bat Sheva, Israel), and CaHA-2 (Radiesse, Merz North America, Inc., Franksville, WI, USA). The panel was purposively selected to include clinically established commercial products from four major categories of long-lasting injectable biostimulators used in aesthetic medicine: PLLA, PDLLA, PCL, and CaHA. More than one product per platform was included to document differences in the appearance of commercial formulations sharing a principal material. The panel encompasses different carriers and presentations and was not designed as a statistically representative market sample. Product identity, manufacturer, formulation/presentation, key formulation context, and reconstitution status are summarized in Table 1. Study codes are used throughout the remainder of the manuscript.

The manufacturer-specified diluent, volume, needle gauge, mixing procedure, and mixing duration were retained for each reconstitution-required product (Table 2). Ready-to-use formulations (PCL-1, PCL-2, CaHA-1, and CaHA-2) were evaluated as supplied, without reconstitution. Manufacturer-specified post-mixing waiting or hydration times are reported in Table 2 for contextual comparison. For the experimental workflow, all reconstitution-required products (PLLA-1 to PLLA-3, PDLLA-1, and PDLLA-2) remained in their original vials through a standardized 120-min post-mixing observation period before baseline microscopy and aqueous exposure. This common interval supported a consistent observation schedule across products and was a study-defined bench condition rather than a recommendation for clinical preparation or administration. All reconstitution-required products were prepared by one physician with clinical experience in the use of the included devices (P.K.).

### 2.2. Macroscopic Evaluation During the Standardized Post-Reconstitution Period

For reconstitution-required products, serial macroscopic observations were performed under standardized lighting and optical conditions immediately after completion of the initial mixing procedure (0 min) and after 30, 60, and 120 min of undisturbed standing. The 0-min assessment represented the immediate post-mixing appearance following addition of the product-specific diluent: sterile 0.9% NaCl for PLLA-1 and sterile water for injection (SWFI) for PLLA-2, PLLA-3, PDLLA-1, and PDLLA-2; it served as a standardized observational timepoint rather than as a harmonized manufacturer-defined end state. These serial vial observations were intended to characterize time-dependent macroscopic changes and did not represent separate administration conditions. After the 120-min observation, each vial was manually re-agitated by the same experienced physician, using routine clinical handling until the contents were judged macroscopically resuspended and suitable for sampling. Assessments were recorded using the predefined descriptive endpoints summarized in Table 3. Ready-to-use formulations did not undergo this serial post-mixing assessment. In the revised reporting, descriptor wording was clarified to emphasize visual appearance and comparisons between unpaired fields; the recorded observations and category assignments were unchanged.

### 2.3. Baseline Light Microscopy

Microscopic assessment was performed using a Levenhuk D95L LCD digital light microscope (Levenhuk, Inc., Tampa, FL, USA) at 40×, 100×, and 400× under standardized optical conditions. For every reconstitution-required product, baseline microscopy was performed after completion of the common 120-min vial-observation period and subsequent manual re-agitation. Immediately thereafter, an aliquot of up to 0.1 mL was withdrawn from the same vial used for serial macroscopic assessment and transferred for microscopy. This study-defined baseline timing was applied uniformly, irrespective of the manufacturer-specified waiting or hydration times summarized in Table 2. For ready-to-use formulations, an aliquot was withdrawn for baseline microscopy directly after opening, after which the remaining syringe contents were expressed into the dish. Baseline observations were recorded using the predefined descriptor sets summarized in Table 3. For each product, three randomly selected microscopic fields were photographed at each magnification at baseline and after 72 h incubation. All acquired fields were assessed; representative fields were selected for presentation in this article.

### 2.4. Simplified Aqueous-Exposure Workflow and Post-Incubation Assessment

After the baseline microscopy sample had been withdrawn, the remaining reconstituted material from each vial was introduced into a standard 9 cm Petri dish (Anumbra, FGH plus s.r.o., Sumperk, Czech Republic) initially containing 15 mL of sterile 0.9% NaCl. For all reconstitution-required products, transfer to the aqueous-exposure workflow therefore occurred at the end of the standardized 120-min vial-observation period. Ready-to-use formulations were expressed directly from the syringe into the dish. Immediate bulk behavior under the applied product-specific deposition conditions was documented macroscopically. The dishes were sealed with Parafilm (Amcor Flexibles North America, Neenah, WI, USA) and incubated for 72 h at 37 °C. This endpoint was selected on the basis of the authors’ clinical observations of early post-treatment swelling during the first approximately three days after injection. It provided a clinically motivated interval for exploring visible morphological differences during aqueous exposure. The study did not evaluate clinical post-treatment edema or its mechanism and did not include intermediate assessments at 24 or 48 h. This acellular exposure did not simulate the cellular, enzymatic, or mechanical conditions of living tissue. Immediately after opening and macroscopic photography, the contents of each dish were mixed with a spatula (Karl Hecht Glaswarenfabrik GmbH & Co KG, Sondheim vor der Rhön, Germany) until a macroscopically homogeneous suspension was obtained. A central sample was then withdrawn using a 1 mL syringe (Becton, Dickinson and Company, Franklin Lakes, NJ, USA) with an 18 G needle (BD Microlance™ 3, Becton Dickinson, Fraga (Huesca), Spain); 0.1 mL was transferred immediately for microscopic assessment. All microscopic sample handling and image acquisition were performed by one experienced laboratory diagnostician (W.G.). The physician (P.K.) and the laboratory diagnostician (W.G.) first assessed the images independently using the predefined descriptor sets summarized in Table 3, then reached consensus on descriptor assignments and qualitative comparisons. Both assessors knew the product identities; the assessment was not blinded. One sample derived from one unit of each product was evaluated. The three fields per product, microscopy stage, and magnification were observations within that sample and were not independent product replicates. No statistical analyses were performed. Baseline and post-incubation fields were unpaired, and the same particles were not tracked longitudinally. 

The manuscript was prepared using Microsoft Word for Microsoft 365 (version 2608, build 16.0.20326.20072, 64-bit; Microsoft Corporation, Redmond, WA, USA). Figures were prepared using Adobe Photoshop (version 27.10; Adobe Inc., San Jose, CA, USA).

## 3. Results

### 3.1. Macroscopic Observations During the Standardized Post-Reconstitution Period

Serial macroscopic observations during the standardized 120-min post-reconstitution period showed visible variation among the examined product units (Figure 1 and Table 4). These observations describe one unit per product under the study-defined timing conditions and do not establish appearance at each manufacturer-recommended administration time. Immediately after mixing (0 min), four of the five reconstitution-required products (PLLA-1, PLLA-3, PDLLA-1, and PDLLA-2) had a single-phase appearance, whereas PLLA-2 had a two-phase liquid-foam appearance. PLLA-1, PLLA-2, and PLLA-3 appeared visually free-flowing, while PDLLA-1 and PDLLA-2 had a visually thick/cohesive appearance. These descriptions were based on subjective visual assessment; no rheological measurements were performed. Surface foam was not observed for PLLA-1, PDLLA-1, or PDLLA-2; it was observed from minute 0 to 120 min for PLLA-2 and at 120 min only for PLLA-3. Wall-associated material was observed from minute 0 to 120 min for PLLA-2, PDLLA-1, and PDLLA-2, but not for PLLA-1 or PLLA-3. During undisturbed standing, PLLA-1 and PLLA-2 showed gradual sedimentation, with increased phase delineation in PLLA-2. PLLA-3 developed subtle surface foam at 120 min, while both PDLLA products showed incomplete wall run-down/creeping.

### 3.2. Bulk Behavior During Simplified Aqueous Exposure and After 72 h Incubation

The study products showed three immediate bulk behavior patterns under the applied product-specific deposition conditions (Table 5). Full-volume homogeneous dispersion was recorded for PLLA-1 and PLLA-3. Full-volume non-homogeneous dispersion was observed for PLLA-2, PDLLA-1, PDLLA-2, and PCL-1, with clusters in PLLA-2 and PDLLA-1, clusters and droplets in PDLLA-2, and strand-like features in PCL-1. PCL-2, CaHA-1, and CaHA-2 retained a rod-like extrusion without immediate dispersion.

After 72 h incubation, PLLA-1 showed a milky full-volume suspension with a small central deposit and lighter streaks, whereas PLLA-3 retained a milky full-volume suspension with mild central clearing. PLLA-2, PDLLA-1, and PDLLA-2 showed a central deposit with a clearer surrounding phase, accompanied by aggregate-like features in PLLA-2 and PDLLA-1 and streak-/band-like heterogeneity in PDLLA-2. PCL-1 showed uniform turbidity and no longer displayed strand-like features. PCL-2, CaHA-1, and CaHA-2 formed swollen localized hydrated masses with visible volume expansion. The initial rod-like configuration was no longer evident in PCL-2; clumps were present in CaHA-1; and CaHA-2 retained a rod-like architecture. Macroscopic bulk behavior is shown in Figure 2.

### 3.3. Baseline Light Microscopy Observations

No clear qualitative differences in the general assessed features were observed among the three recorded fields for any given product, microscopy stage, and magnification. Baseline light microscopy identified four dominant morphological categories across the examined samples: spherical particles, ovoid particles, irregular particles, and spindle-like microstructures (Table 6 and Figure 3). Within the PLLA platform, PLLA-1 showed spherical particles with a uniform apparent optical appearance and no aggregation, whereas PLLA-2 and PLLA-3 showed irregular particles with non-uniform apparent optical appearances and moderate and marked aggregation, respectively. Bubble-associated clustering was recorded only for PLLA-2. Both PDLLA products showed ovoid particle populations with a non-uniform apparent optical appearance, a subset of deformed particles, and slight aggregation. PCL-2, CaHA-1, and CaHA-2 showed spherical particle populations with a mostly uniform apparent optical appearance and no aggregation. Additional baseline features included a deformed particle subset in PCL-2, apparent surface depressions in CaHA-1, and a deformed subset with doublet forms in CaHA-2. PCL-1 showed spindle-like microstructures with a non-uniform apparent optical appearance and moderate aggregation rather than the spherical particles observed in the other ready-to-use products.

### 3.4. Post-Incubation Light Microscopy Observations Relative to Baseline

After 72 h incubation, the assessed fields for PLLA-1, PLLA-3, CaHA-1, and CaHA-2 had the same dominant morphology and qualitative aggregation categories as the baseline fields, with no clear particle or microstructure difference detected (Table 7). No additional microscopic feature was recorded for PLLA-1. Small aggregates were present in post-incubation fields for PLLA-3; apparent surface depressions were observed at both stages for CaHA-1; and deformed profiles with doublet forms were observed at both stages for CaHA-2. PLLA-2 had the irregular morphology category at both stages, with no clear particle difference detected and a lower qualitative aggregation category after incubation.

Both PDLLA samples had the ovoid morphology category at both stages. Post-incubation fields were assigned subtle apparent morphological differences and a higher aggregation category relative to baseline, with crescent/bean-like forms observed after incubation. PCL-1 had the spindle-like microstructural category at both stages; post-incubation fields were assigned marked apparent differences and a lower aggregation category and contained markedly shorter microstructures. PCL-2 had the spherical morphology category at both stages and was assigned subtle apparent differences with the same aggregation category; deformed profiles recorded at baseline were not identified in post-incubation fields (Figure 4). These descriptions compare unpaired fields and do not demonstrate transformation of individual particles.

## 4. Discussion

This pilot study documented macroscopic and light microscopic features of samples from one unit of each of nine commercial biostimulator products. The recorded descriptors did not align uniformly with nominal material platform: some samples within a platform differed, while samples across platforms shared selected features. Product-level descriptive reporting therefore adds information to the platform classification, although these observations do not establish reproducibility or physicochemical non-equivalence.

Previous comparative studies examined narrower product groups using physicochemical or morphological endpoints [7,8,9,11]. The contribution of the present study is a common descriptive sequence spanning four platforms and both reconstitution-required and ready-to-use presentations. Table 1 and Table 2 provide product and preparation context, Table 3 defines the descriptive framework, and Table 4, Table 5, Table 6 and Table 7 and the figures report the authors’ observations. The comparison concerns complete commercial formulations under the stated conditions and does not isolate the effect of the principal material.

Serial vial observations documented visual appearance, flow, foam, wall-associated material, and changes during standing. The two PDLLA samples shared a visually thick/cohesive appearance; this was a subjective observation, not a rheological measurement. Preparation and handling have also been examined in comparative PLA/PDLLA studies [8,9]. For the five reconstitution-required products, product-specific diluents and mixing procedures were retained, while the interval before re-agitation and baseline microscopy was fixed at 120 min. This common interval may have influenced sedimentation, hydration, aggregation, and microscopic appearance, and its influence may differ between formulations. Because no comparison with every manufacturer-specific administration time was performed, the results cannot establish equivalent preparation states across products. The 120-min interval is an experimental condition and should not be adopted as clinical preparation guidance; clinical preparation should follow the applicable product IFU.

The bulk observations describe the complete formulations under product-specific deposition conditions. PCL-2, CaHA-1, and CaHA-2 initially retained rod-like extrusions and subsequently formed localized hydrated masses, whereas PCL-1 showed strand-like features followed by uniform turbidity. Among reconstituted samples, PLLA-1 and PLLA-3 dispersed homogeneously, while PLLA-2 and the PDLLA samples showed non-homogeneous dispersion. These observations identify patterns for further investigation. They cannot establish whether the principal material, carrier, concentration, preparation, or deposition method caused a given pattern because these factors were not independently controlled, nor do macroscopic swelling or dispersion in saline demonstrate tissue distribution, clinical edema, material dissolution, or degradation.

Baseline microscopy documented dominant morphology, apparent optical appearance, observed aggregation category, bubble association, and additional visible features. These descriptors distinguished the examined samples beyond their nominal platform or broad morphology category. The PLLA samples differed in morphology and aggregation, while the spherical samples differed in the presence of deformed profiles, apparent surface depressions, or doublet forms. PCL-1 showed spindle-like microstructures.

Published comparative work has likewise reported differences in particle morphology between injectable products [7,8,9]. In the present study, however, apparent optical features remain descriptions of light microscopy images. An empty-interior appearance or apparent surface depression does not establish porosity or three-dimensional surface topography. Similarly, the spindle-like structures observed in PCL-1 do not establish the chemical identity or physical organization of each visible structure.

The light microscopy component provides a preliminary visual reference for investigators who will search for and describe these materials in future tissue sections. Histopathological evaluation can consider both the appearance of implanted material and the surrounding tissue response [10]. The present descriptive method addresses the initial documentation of general particle and microstructure appearance. Whether these features remain recognizable after fixation, embedding, sectioning, staining, and in vivo exposure must be evaluated separately. No cellular response, immunological activity, or clinical efficacy was assessed here.

The dominant morphology categories in post-incubation fields corresponded to baseline across the examined samples, while additional appearances and aggregation categories differed. Crescent/bean-like forms were recorded in both PDLLA samples after incubation; PCL-1 fields contained shorter spindle-like microstructures; and deformed PCL-2 profiles recorded at baseline were not identified after incubation. These are comparisons of unpaired fields. They do not demonstrate transformation, loss, or recovery of individual particles. An absence of clear optical differences does not establish chemical stability. Reporting the specific observed features alongside the broad morphology and aggregation categories preserves this distinction.

This completed descriptive stage identifies questions for two complementary lines of further research. Physicochemical studies should use independent product units and appropriately sampled batches, controlled preparation and exposure conditions, and quantitative endpoints. Depending on the question, these may include particle size and circularity, aggregation metrics, rheology, high-resolution imaging, chemical composition, crystallinity, or molecular-weight measurements. Comparisons of manufacturer-specific and common preparation intervals, together with additional incubation timepoints, would help separate timing effects from formulation differences.

In vivo studies with histopathological assessment would address material appearance and tissue response in a biological environment. The present images may assist the preparation of those studies by documenting general appearances before tissue exposure. The correspondence between these bench observations and processed tissue sections requires direct evaluation. This sequence of studies would extend the descriptive reference without treating it as a surrogate for physicochemical measurements or biological assays.

### Limitations

This pilot study examined one sample from one unit of each product. It cannot estimate within-batch or between-batch variability, establish reproducibility, or support inferential comparisons between products. Three randomly selected microscopic fields were assessed for each product, stage, and magnification, but these observations do not replace independent product replicates or exclude unsampled heterogeneity. The two assessors evaluated images independently before consensus, with knowledge of product identities; the lack of blinding introduces a potential source of observer bias.

Microscopy was qualitative, and baseline and post-incubation fields were unpaired. Individual particles were not tracked. No morphometric quantification, automated image analysis, rheological measurements, scanning electron microscopy, crystallinity analysis, molecular-weight tracking, or other quantitative physicochemical assays were performed. Optical appearance therefore cannot establish particle transformation, surface topography, chemical degradation, or chemical stability.

Preparation and aqueous exposure were not designed to isolate individual formulation variables. Diluents, mixing procedures, transferred quantities, and deposition conditions varied by product; final volumes, concentrations, and ionic compositions were not normalized. For the reconstitution-required products, the common 120-min interval did not reproduce every manufacturer-specific administration schedule, and its effects were not separately tested. Manual re-agitation and post-incubation spatula mixing used operator-judged endpoints. Post-incubation microscopy was based on a central 0.1 mL sample collected after mixing, so residual spatial heterogeneity cannot be excluded.

Only one incubation endpoint, 72 h, was examined; changes at intermediate times and their kinetics cannot be determined. The saline exposure was acellular and did not reproduce tissue confinement, protein adsorption, cellular interactions, enzymatic activity, mechanical forces, massage, or in vivo clearance. It was not a model of post-treatment edema, and the findings do not establish clinical efficacy, safety, or immune responses.

## 5. Conclusions

This descriptive pilot documented macroscopic and light microscopic appearances in samples from one unit of each of nine injectable biostimulator products. The observed descriptors did not align uniformly with nominal material platform, and samples sharing a broad morphology category differed in additional visible features. The design does not establish reproducibility or isolate the effects of material, carrier, preparation, or deposition conditions.

The resulting visual documentation provides a preliminary reference for further physicochemical characterization and the planning of in vivo studies with histopathological assessment. It does not establish chemical degradation or stability, biological effects, clinical performance, or validated product identification in tissue.

## Figures and Tables

**Figure 1:**
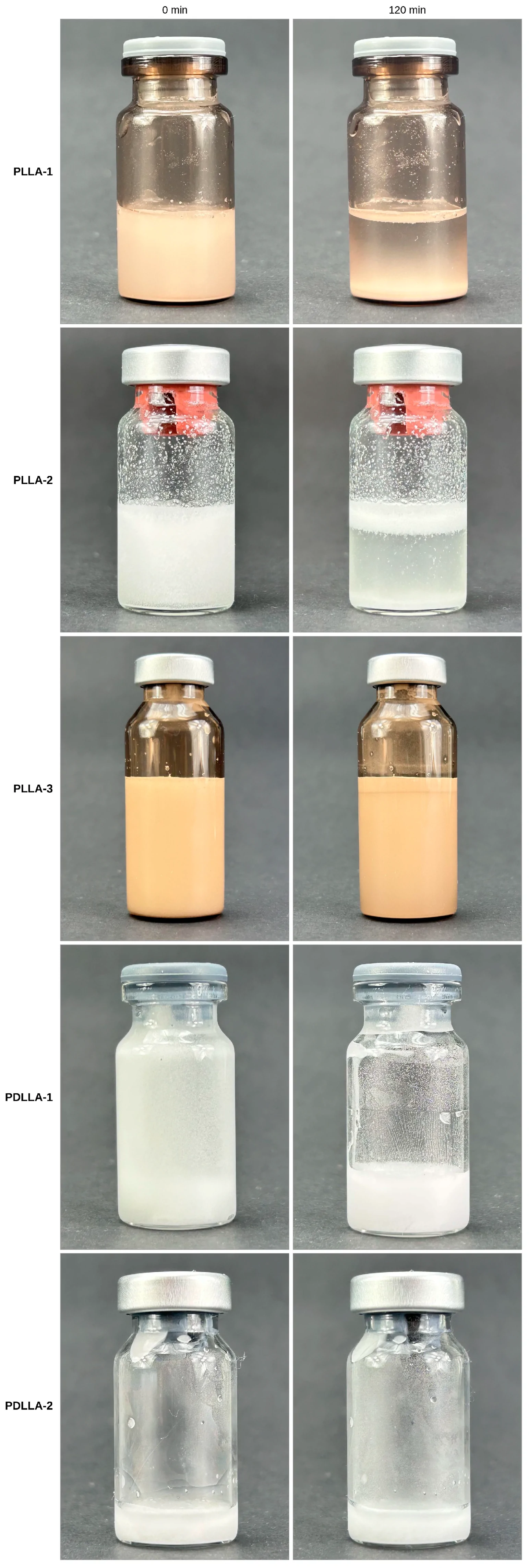
Macroscopic appearance of reconstitution-required products immediately after mixing (0 min) and after 120 min of undisturbed standing.

**Figure 2:**
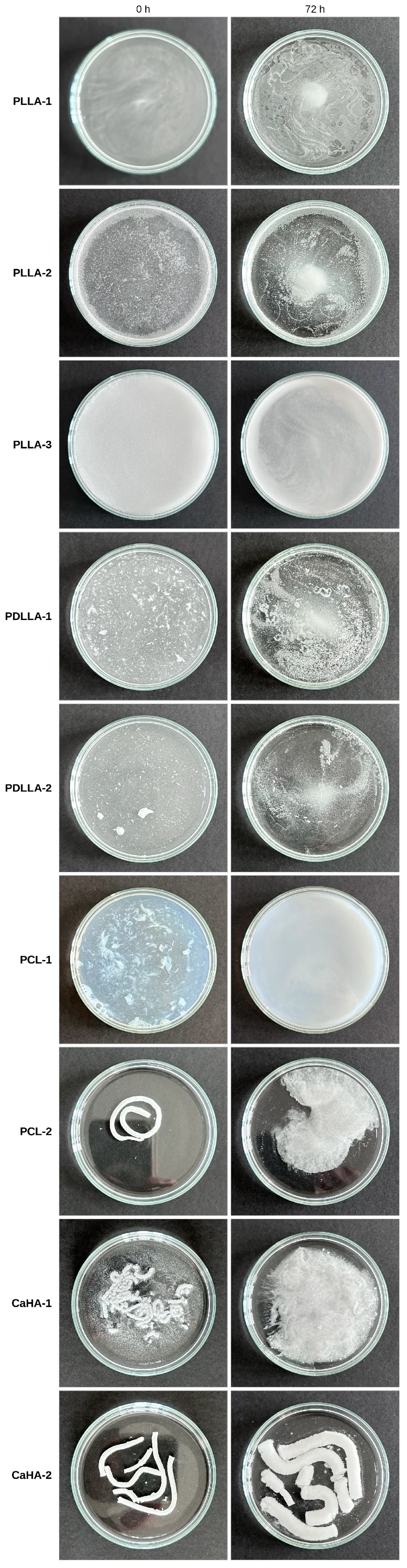
Macroscopic bulk behavior immediately after introduction (0 h) into dishes initially containing 15 mL of sterile 0.9% NaCl and after 72 h incubation, before post-incubation spatula mixing. Images show the complete formulations under the applied product-specific deposition conditions; final product concentrations, volumes, and ionic compositions were not normalized.

**Figure 3:**
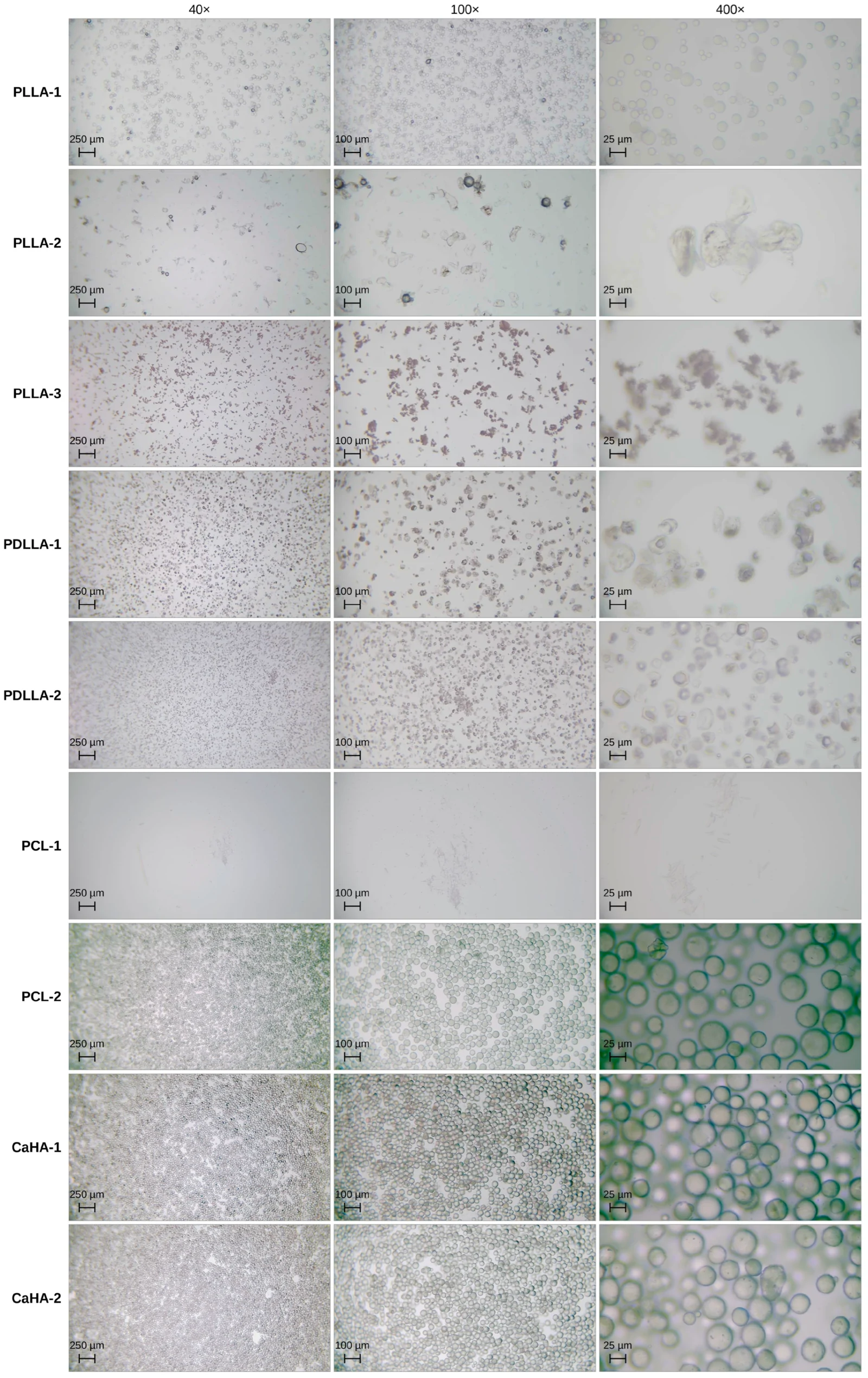
Baseline light microscopy appearance across study products and magnifications. Representative images were selected from three randomly chosen fields per product and magnification, obtained from one unit per product. Baseline and post-incubation fields are unpaired and do not represent longitudinal tracking of the same particles.

**Figure 4:**
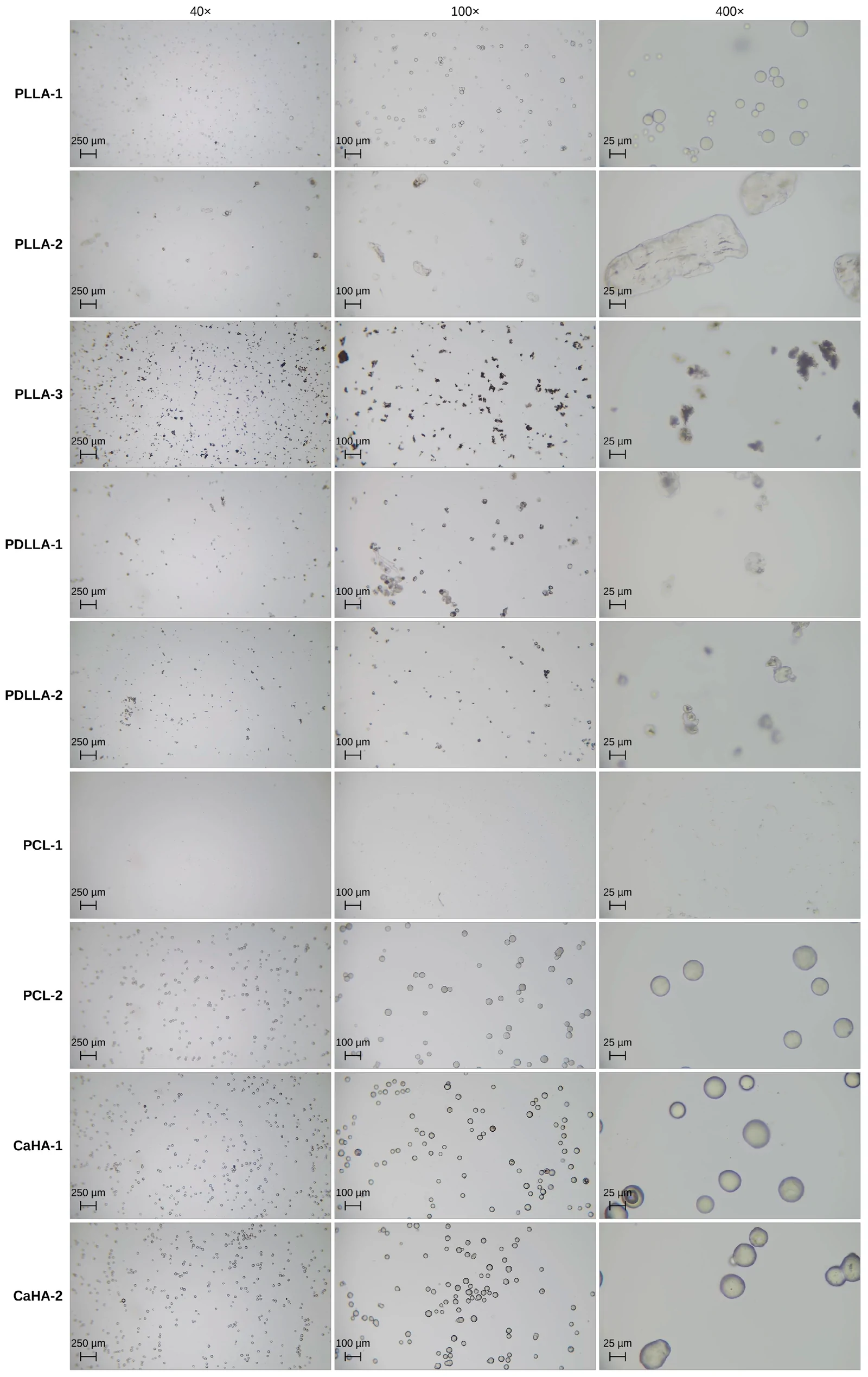
Light microscopy appearance across study products and magnifications after 72 h incubation. Representative images were selected from three randomly chosen fields per product and magnification, obtained from one unit per product. The post-incubation fields are unpaired with baseline fields and do not represent longitudinal tracking of the same particles.

**Table 1:** Commercial injectable biomaterial products included in the pilot bench study and assigned study codes. Formulation entries provide product context and were not independently verified by compositional analysis.

Study Product	Commercial Product (Manufacturer, City, Country)	Principal Material Platform	Formulation/ Presentation	Key Formulation Context	Reconstitution Required
PLLA-1	JULÄINE (Nordberg Medical AB, Huddinge, Sweden)	PLLA	Lyophilized powder for reconstitution	PLLA with CMC and mannitol	Yes
PLLA-2	Sculptra (Galderma, Lausanne, Switzerland)	PLLA	Lyophilized powder for reconstitution	PLLA with CMC and mannitol	Yes
PLLA-3	Lanluma (GANA R&D Co, Ltd., Seongnam-si, Republic of Korea)	PLLA	Lyophilized powder for reconstitution	PLLA with CMC and mannitol	Yes
PDLLA-1	Lenisna 200 (VAIM Co., Ltd., Daejeon, Republic of Korea)	PDLLA	Lyophilized powder for reconstitution	PDLLA with non-cross-linked HA	Yes
PDLLA-2	JUVElook (VAIM Co., Ltd., Daejeon, Republic of Korea)	PDLLA	Lyophilized powder for reconstitution	PDLLA with non-cross-linked HA	Yes
PCL-1	Gouri (Dexlevo Inc., Seoul, Republic of Korea)	PCL	Ready-to-use prefilled syringe	Solubilized PCL in a proprietary liquid carrier	No
PCL-2	Ellansé (AQTIS Medical BV, Utrecht, The Netherlands)	PCL	Ready-to-use prefilled syringe	PCL microspheres in an aqueous CMC gel	No
CaHA-1	HArmonyCa (Panaxia, Ltd., Bat Sheva, Israel)	CaHA	Ready-to-use prefilled syringe	CaHA microspheres with cross-linked HA in an aqueous hybrid gel	No
CaHA-2	Radiesse (Merz North America, Inc., Franksville, WI, USA)	CaHA	Ready-to-use prefilled syringe	CaHA microspheres with a CMC gel carrier	No

Abbreviations: CMC, carboxymethylcellulose; HA, hyaluronic acid.

**Table 2:** Manufacturer-specified preparation parameters for reconstitution-required products.

Study Product	Diluent Type	Diluent Volume	Needle Gauge	Mixing Procedure	Mixing Duration	Manufacturer-Specified Post-Mixing Waiting/Hydration Time
PLLA-1	Sterile 0.9% NaCl	5 mL	18 G	Gentle rotation/inversion after diluent addition	~30 s	2 h standing
PLLA-2	Sterile water for injection (SWFI)	5 mL	18 G	Vigorous shaking after diluent addition	~3 min	2 h hydration
PLLA-3	Sterile water for injection (SWFI)	15 mL	18 G	Thorough shaking after diluent addition	10 min	1 h hydration
PDLLA-1	Sterile water for injection (SWFI)	3 mL	18 G	Vortex mixing in two cycles separated by a 5 min rest period	3 min + 3 min	None
PDLLA-2	Sterile water for injection (SWFI)	0.75 mL	18 G	Vortex mixing in two cycles separated by a 5 min rest period	3 min + 3 min	None

Abbreviations: NaCl, sodium chloride; SWFI, sterile water for injection. Note: Table 2 reports manufacturer-specified preparation parameters for contextual comparison. In the experimental workflow, all reconstitution-required products underwent a standardized 120-min vial-observation interval; each vial was then manually re-agitated, an aliquot of up to 0.1 mL was withdrawn for baseline microscopy, and the remaining material was transferred to the aqueous-exposure workflow. This study-defined sequence should not be interpreted as a manufacturer-recommended administration schedule.

**Table 3:** Predefined descriptive endpoints and interpretive boundaries for the pilot observational workflow.

Observation Stage	Endpoint	Permitted Descriptor Set	Interpretive Boundary
Standardized post-reconstitution vial observation (reconstitution-required products only)	Appearance immediately after mixing (0 min)	Single-phase suspension; two-phase liquid-foam appearance	Descriptive only; not a validated measure of formulation homogeneity
	Visual consistency immediately after mixing (0 min)	Visually free-flowing; visually thick/cohesive	Subjective visual assessment of appearance and flow; viscosity and viscoelastic moduli were not measured
	Notable preparation feature(s)	Surface foam observed/not observed; wall-associated material observed/not observed	Feature annotation only
	Change during undisturbed standing (30–120 min)	Gradual sedimentation; increased phase delineation; incomplete wall run-down/creeping; subtle surface foam	Descriptive only; not a formal sedimentation or hydration-kinetics analysis
Baseline light microscopy	Dominant morphology category	Spherical; ovoid; irregular; spindle-like microstructures	Descriptive morphological grouping only
	Apparent optical appearance	Uniform; mostly uniform; non-uniform	Qualitative light microscopy appearance only; surface topography and porosity were not measured
	Observed aggregation category	None; slight; moderate; marked	Qualitative visual category; no quantitative cluster analysis. “None” means no aggregation was recorded in the assessed fields
	Bubble association	No visible bubbles; rare bubbles; bubbles present; bubble-associated clustering observed	Descriptive only; visible co-occurrence of particles and bubbles, not gas quantification
	Notable baseline feature(s)	No additional notable feature; deformed particles; apparent surface depressions; paired/doublet forms; empty-interior appearance; elongated microstructures; sharp-edged particles	Feature annotation only
Early aqueous exposure	Immediate bulk behavior	Full-volume homogeneous dispersion; full-volume non-homogeneous dispersion; retained rod-like extrusion/no immediate dispersion; clusters; droplets; strand-like features	Simplified bench model; observed behavior reflects the complete formulation, preceding preparation context, and product-specific deposition conditions and cannot be attributed to any single variable
72 h macroscopic assessment	72 h bulk appearance	Milky full-volume suspension; milky full-volume suspension with small central deposit; central deposit with clearer surrounding phase; swollen localized hydrated mass; uniform turbidity	Descriptive bulk outcome only; not a formal stability claim
	Additional macroscopic observation(s)	Aggregate-like features; lighter streaks; streak-/band-like heterogeneity; clumps; mild central clearing; volume expansion; loss or retention of rod-like configuration; loss of strand-like features	Feature annotation only
72 h light microscopy	Dominant morphology after 72 h	Spherical; ovoid; irregular; spindle-like microstructures	Descriptive morphological grouping relative to baseline
	Apparent particle/microstructure differences between unpaired fields	No clear difference detected; subtle apparent differences; marked apparent differences	Qualitative comparison of unpaired fields; individual particles were not tracked. These descriptors do not establish particle transformation, dissolution, or chemical degradation
	Aggregation category relative to baseline fields	Same category; lower category; higher category	Qualitative category comparison between unpaired fields; no particle counts, aggregate-area measurements, or inferential analysis
	Additional microscopic observation(s)	No additional notable feature; small aggregates present in post-incubation fields; crescent/bean-like forms observed in post-incubation fields; microstructures markedly shorter than in baseline fields; deformed forms recorded at baseline not identified in post-incubation fields; apparent surface depressions observed in both baseline and post-incubation fields; deformed subset and doublet forms observed in both baseline and post-incubation fields	Feature annotation only

**Table 4:** Summary of immediate and time-dependent macroscopic observations during the standardized 120-min post-reconstitution period.

Study Product	Appearance Immediately After Mixing (0 min)	Visual Consistency Immediately After Mixing (0 min)	Surface Foam During 0–120 min	Wall-Associated Material During 0–120 min	Change During Standing (30–120 min)
PLLA-1	Single-phase suspension	Visually free-flowing	Not observed	Not observed	Gradual sedimentation
PLLA-2	Two-phase liquid-foam appearance	Visually free-flowing	Observed from minute 0	Observed from minute 0	Gradual sedimentation; increased phase delineation
PLLA-3	Single-phase suspension	Visually free-flowing	Observed at 120 min only	Not observed	Subtle surface foam at 120 min
PDLLA-1	Single-phase suspension	Visually thick/cohesive	Not observed	Observed from minute 0	Incomplete wall run-down/creeping
PDLLA-2	Single-phase suspension	Visually thick/cohesive	Not observed	Observed from minute 0	Incomplete wall run-down/creeping

**Table 5:** Descriptive bulk behavior under the applied product-specific deposition conditions and after 72 h incubation.

Study Product	Immediate Bulk Behavior Under the Applied Product-Specific Deposition Conditions	72 h Bulk Appearance	Additional Macroscopic Observation(s) After 72 h
PLLA-1	Full-volume homogeneous dispersion	Milky full-volume suspension with small central deposit	Lighter streaks present
PLLA-2	Full-volume non-homogeneous dispersion with clusters	Central deposit with clearer surrounding phase	Aggregate-like features present
PLLA-3	Full-volume homogeneous dispersion	Milky full-volume suspension	Mild central clearing
PDLLA-1	Full-volume non-homogeneous dispersion with clusters	Central deposit with clearer surrounding phase	Aggregate-like features present
PDLLA-2	Full-volume non-homogeneous dispersion with clusters and droplets	Central deposit with clearer surrounding phase	Streak-/band-like heterogeneity
PCL-1	Full-volume non-homogeneous dispersion with strand-like features	Uniform turbidity	Strand-like features no longer evident
PCL-2	Retained rod-like extrusion/no immediate dispersion	Swollen localized hydrated mass	Initial rod-like configuration no longer evident; volume expansion
CaHA-1	Retained rod-like extrusion/no immediate dispersion	Swollen localized hydrated mass	Volume expansion; clumps present
CaHA-2	Retained rod-like extrusion/no immediate dispersion	Swollen localized hydrated mass	Volume expansion; retained rod-like architecture

**Table 6:** Baseline light microscopy descriptors obtained after completion of the standardized 120-min vial-observation period and subsequent re-agitation for reconstitution-required products, and directly after opening for ready-to-use products.

Study Product	Dominant Morphology	Apparent Optical Appearance	Observed Aggregation Category	Bubble Association	Notable Baseline Feature(s)
PLLA-1	Spherical particles	Uniform	None	Rare bubbles	No additional notable feature
PLLA-2	Irregular particles	Non-uniform	Moderate	Bubbles present; bubble-associated clustering observed	Predominantly sharp-edged particles
PLLA-3	Irregular particles	Non-uniform	Marked	No visible bubbles	Empty-interior appearance in a subset of particles; sharp-edged particles
PDLLA-1	Ovoid particles	Non-uniform	Slight	No visible bubbles	Subset of particles deformed
PDLLA-2	Ovoid particles	Non-uniform	Slight	No visible bubbles	Subset of particles deformed
PCL-1	Spindle-like microstructures	Non-uniform	Moderate	No visible bubbles	Elongated microstructures; sharp-edged particles
PCL-2	Spherical particles	Mostly uniform	None	No visible bubbles	Subset of particles deformed
CaHA-1	Spherical particles	Mostly uniform	None	No visible bubbles	Apparent surface depressions of variable size
CaHA-2	Spherical particles	Mostly uniform	None	No visible bubbles	Subset of particles deformed; doublet forms observed

**Table 7:** Light microscopy descriptors after 72 h incubation relative to baseline, based on qualitative assessment of three randomly selected fields per product, microscopy stage, and magnification. Baseline and post-incubation fields were unpaired. Category labels describe apparent differences between fields, without longitudinal tracking of individual particles or quantitative assessment of aggregation.

Study Product	Dominant Morphology After 72 h	Apparent Particle/ Microstructure Differences Between Unpaired Fields	Aggregation Category Relative to Baseline Fields	Additional Microscopic Observation(s)
PLLA-1	Spherical particles	No clear difference detected	Same category	No additional notable feature
PLLA-2	Irregular particles	No clear difference detected	Lower category	No additional notable feature
PLLA-3	Irregular particles	No clear difference detected	Same category	Small aggregates present in assessed post-incubation fields
PDLLA-1	Ovoid particles	Subtle apparent differences	Higher category	Crescent/bean-like forms observed in assessed post-incubation fields
PDLLA-2	Ovoid particles	Subtle apparent differences	Higher category	Crescent/bean-like forms observed in assessed post-incubation fields
PCL-1	Spindle-like microstructures	Marked apparent differences	Lower category	Microstructures markedly shorter than in assessed baseline fields
PCL-2	Spherical particles	Subtle apparent differences	Same category	Deformed forms recorded in assessed baseline fields not identified in assessed post-incubation fields
CaHA-1	Spherical particles	No clear difference detected	Same category	Apparent surface depressions observed in both assessed baseline and post-incubation fields
CaHA-2	Spherical particles	No clear difference detected	Same category	Deformed subset and doublet forms observed in both assessed baseline and post-incubation fields

## Data Availability

The data presented in this study are available upon request from the corresponding author. The data are not publicly available because they constitute the intellectual property of K-LAB Badania i Rozwój, Gdynia, Poland.
